# The Impact of Preoperative Risk Factors on Unplanned Readmission After Day Surgery: A Meta-Analysis

**DOI:** 10.3390/jpm15070281

**Published:** 2025-07-01

**Authors:** Hanqing Zhang, Xinglian Gao, Zhen Chen

**Affiliations:** 1 Union Hospital, Tongji Medical College, Huazhong University of Science and Technology, Wuhan 430030, China; m202375993@hust.edu.cn; 2 School of Nursing, Tongji Medical College, Huazhong University of Science and Technology, Wuhan 430030, China; 3 Eye Center, Renmin Hospital of Wuhan University, Wuhan 430060, China; hchenzhen@163.com

**Keywords:** day surgery, preoperative risk factors, meta-analysis, unexpected readmissions

## Abstract

**Objective**: This research seeks to explore and determine the principal pre-surgical risk elements associated with unplanned readmissions following day surgery, providing evidence-based guidance for clinical practice to optimize preoperative evaluations and reduce the incidence of readmissions. **Background**: As day surgery becomes increasingly common across global healthcare systems, ensuring effective postoperative recovery and preventing readmissions have become critical challenges. Numerous studies have explored the impact of various preoperative risk factors on postoperative readmissions. This study synthesizes existing evidence through a meta-analysis to identify the key preoperative factors associated with increased readmission risk. **Methods**: An extensive literature review was conducted across various databases, such as Web of Science, PubMed, CINAHL, Scopus, Embase, the Cochrane Library, and CNKI, to gather all relevant clinical research on pre-surgical risk elements for day surgery procedures, with studies included up to 15 January 2025. A structured analysis was undertaken, and the findings were integrated using a random-effects approach to assess the influence of key preoperative risk factors on subsequent readmissions in day surgery environments. **Results**: A total of 12 studies, involving 704,568 patients, were incorporated into the final analysis. The findings identified several preoperative factors that were significantly associated with an increased risk of postoperative readmission. These risk factors included: age ≥ 60 years (OR = 2.38, 95% CI: 1.74–3.26, *p* < 0.00001), ASA classification ≥ 3 (OR = 1.96, 95% CI: 1.61–2.38, *p* < 0.00001), presence of chronic diseases (OR = 11.78, 95% CI: 9.99–13.90, *p* < 0.00001), general anesthesia (OR = 2.42, 95% CI: 1.51–3.86, *p* = 0.0002), infection risk (OR = 1.68, 95% CI: 1.35–2.10, *p* < 0.00001), gender (OR = 2.45, 95% CI: 2.21–2.71, *p* < 0.00001), complex surgery type (OR = 2.83, 95% CI: 2.03–3.93, *p* < 0.00001), and bleeding disorders (OR = 1.82, 95% CI: 1.53–2.17, *p* < 0.00001). **Conclusions**: This study highlights several key preoperative risk factors associated with unexpected readmissions following day surgery. These factors include age, ASA classification, presence of chronic diseases, general anesthesia, infection risk, gender, complex surgery type, and bleeding disorders. These findings provide valuable insights for preoperative assessments. Clinicians should focus on these high-risk factors during preoperative assessment and management to minimize postoperative readmission rates and improve surgical safety and recovery outcomes for patients.

## 1. Introduction

Day surgery, characterized by a same-day discharge, has become a cornerstone of modern healthcare due to its efficiency and cost-effectiveness. However, its rapid discharge model renders patients particularly vulnerable to unplanned readmissions. The abbreviated hospital stay limits postoperative monitoring, potentially overlooking early complications such as infections or bleeding, which may necessitate readmission [1,2,3]. Additionally, patients recover at home with minimal professional oversight, increasing the risk of non-compliance with care plans or delayed recognition of adverse events [4]. This vulnerability underscores the need to identify preoperative risk factors to enhance patient selection and reduce readmission rates in this high-risk population.

With the continuous development of medical care models, day surgery has become a progressively common approach to care. Its notable benefits include reducing patient hospitalization time, lowering medical costs, decreasing the risk of postoperative infections, and improving hospital bed turnover rates [1,2]. Readmissions not only increase the physical and mental burden on patients and affect the quality of postoperative recovery, but also incur higher medical costs [3]. Therefore, reducing the rate of postoperative readmissions is an important issue that needs to be addressed in day surgery. Preoperative risk assessment plays a crucial role in determining the suitability of patients for day surgery. A thorough preoperative evaluation allows healthcare providers to identify and address potential risk factors that could influence surgical outcomes and postoperative recovery, including an advanced age, chronic medical conditions, and coagulation abnormalities [4,5,6].

Despite existing studies exploring numerous preoperative risk factors, the findings are inconsistent due to variations in the study designs, participant demographics, and analytical approaches. For example, when assessing the influence of age and ASA classification on postoperative readmission following day surgery, some research identifies an advanced age and ASA ≥ 3 as significant risk factors for postoperative recovery, while other studies fail to find a significant association [7,8]. Moreover, general anesthesia, infection risk, and presence of chronic diseases have been identified as key risk factors in certain investigations, but their applicability across diverse surgical procedures and patient populations has yet to be fully validated [9,10,11].

Despite existing studies exploring numerous preoperative risk factors, findings remain inconsistent, limiting clarity on which factors most strongly predict unplanned readmission [7,8,9,10,11]. For example, some studies link age and ASA classification to a higher readmission risk, while others find no association [7,8]. Moreover, general anesthesia, infection risk, and chronic diseases have been identified as risk factors in certain investigations, but their relative importance across diverse procedures and populations remains unclear [9,10,11].

Therefore, this meta-analysis addresses the gap in understanding which preoperative risk factors most strongly predict unplanned readmission following day surgery. By synthesizing robust evidence, it aims to identify the preoperative factors with a significant predictive impact on readmission risk, offering scientific guidance to support clinicians in optimizing preoperative assessments and management strategies, reducing postoperative readmission rates, improving healthcare quality, enhancing postoperative recovery, and increasing patient satisfaction.

## 2. Methods

### 2.1. Registration ID

The review protocol was officially registered with the International Prospective Register of Systematic Reviews (PROSPERO) under the identification number CRD42024602853. This systematic review was carried out following the guidelines outlined in the Preferred Reporting Items for Systematic Reviews and Meta-Analyses (PRISMA) statement, as well as adhering to the standards recommended by the Meta-analysis of Observational Studies in Epidemiology (MOOSE) framework.

### 2.2. Retrieval Methodology

Two authors independently performed an extensive review of the literature using electronic databases. The search encompassed studies published from the inception of each database up to 15 January 2025, and targeted research examining the influence of preoperative risk factors on postoperative hospital readmissions following day surgery. The databases consulted included Web of Science, PubMed, CINAHL, Scopus, Embase, the Cochrane Library, and CNKI. The search keywords included “Preoperative risk factors” OR “Preoperative assessment” OR “Preoperative evaluation” OR “Preoperative factors” OR “Pre-surgical risk factors” OR “Pre-surgical assessment” OR “Pre-surgical evaluation” AND “Day surgery” OR “Ambulatory surgery” OR “Same-day surgery” OR “Day-case surgery” OR “Day case surgery” OR “Day operation” OR “Day-operation” OR “Day surgeries” OR “Day-surgery” AND “Hospital readmission” OR “Readmission” OR “Unplanned readmission” OR “Rehospitalization”. The retrieval approach integrated both Medical Subject Headings (MeSH) and free-text keywords, and terms were flexibly combined to maximize retrieval sensitivity. The query was confined to articles released in the English language, but no limitations were imposed concerning the year of publication, geographical setting, or study type. Additionally, the reference lists of all qualifying articles were carefully reviewed to uncover further pertinent studies.

### 2.3. Selection and Exclusion Standards

Research was selected according to the following standards: (1) Population: Individuals undergoing day surgery procedures; (2) Research design: Observational studies, specifically cohort or case–control designs, that explored preoperative risk factors related to day surgery; (3) Outcomes: Factors such as patient age, ASA physical status classification, existing chronic illnesses, general anesthesia, infection risk, gender, surgery type, presence of bleeding disorders, etc.; (4) Data reporting: Studies that provided odds ratios (ORs), 95% confidence intervals (CIs), or other relevant statistical indicators. Exclusion criteria encompassed the following: (1) Study design: Non-original research including fundamental clinical trials, opinion pieces, studies on animals, narrative reviews, case studies, republished materials, or symposium proceedings; (2) Information integrity: Studies with missing or inaccurate data, statistical inconsistencies, incorrect citations, or duplicated publications; (3) Access: Unavailable sources.

### 2.4. Information Retrieval and Compilation

Grounded in predefined selection and elimination guidelines, a pair of researchers initially reviewed a total of 23,791 articles. Reference management was conducted using EndNote 21 software. Following the removal of duplicate records, the titles and abstracts were independently evaluated by both authors, with full texts subsequently assessed for eligibility. Data extraction was carried out systematically, and its accuracy was confirmed by an impartial additional researcher. When disagreements arose, a senior researcher was engaged to achieve a mutual agreement. Extracted data from each eligible study included the subsequent details: basic data (primary author, publication year, and country of origin), research structure (study type, participant count, and inclusion standards), demographic and clinical characteristics of patients (age and sex), surgical and anesthetic details (type and duration of surgery, as well as anesthesia approach), and identified preoperative risk factors (age, ASA classification, presence of chronic diseases, general anesthesia, infection risk, gender, complex surgery type, bleeding disorders, etc). Additionally, when accessible, adjusted odds ratios (ORs) and their 95% confidence intervals (CIs) obtained from multivariable regression models were documented, together with the variables incorporated in those analyses. In cases where essential information was missing or ambiguous, the corresponding authors were contacted for clarification. Ultimately, 12 studies satisfied the standards and were incorporated into the ultimate evaluation.

### 2.5. Quality Assessment

To ensure methodological rigor, two authors evaluated the standard of the included research. The Newcastle–Ottawa Scale (NOS) was employed to evaluate both case–control and cohort research designs, which comprises three major domains: (1) Selection (consisting of four component criteria), (2) Comparability (one component criteria), and (3) Outcome (three component criteria). Each sub-item under Selection and Outcome domains was assigned a maximum of one point, while the Comparability criterion could contribute up to two points, making the total achievable score nine. According to the total score, research was classified into three quality tiers: low (0–3 points), medium (4–5 points), and high (6–9 points). Differences among the researchers were settled through deliberation, involving a third researcher’s input when initial agreement was not achieved.

### 2.6. Data Assessment

This meta-analysis was conducted with Review Manager (RevMan) software, version 5.4, created by the Cochrane Collaboration. For continuous outcomes, effect sizes were estimated using either the mean difference (MD) or the standardized mean difference (SMD) in the evaluation process. In the case of dichotomous outcomes, effect sizes were represented by the odds ratio (OR) or the relative risk (RR), each with a corresponding 95% confidence interval (CI). To evaluate variability among the included studies, the I^2^ statistic was employed. An I^2^ > 50% indicated considerable heterogeneity, prompting further investigation through subgroup and sensitivity analyses to identify and address potential sources of variation. Conversely, if the I^2^ < 50%, heterogeneity was considered low, and a fixed-effect model (FEM) was subsequently adopted. Statistical significance was established using a threshold of *p* < 0.05.

## 3. Results

### 3.1. Literature Selection

**Figure 1:**
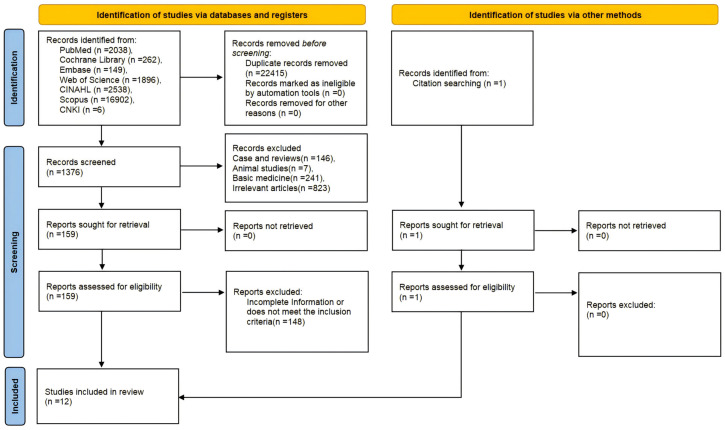
PRISMA flowchart of study selection: 23,791 records screened and 12 studies included.

Using the established search protocol, an initial total of 23,791 entries were retrieved. Following an initial screening, 1376 studies were deemed potentially relevant. After carefully reviewing the titles and abstracts, inclusion and exclusion criteria were applied. A thorough review of the full texts resulted in the final selection of 12 articles [12,13,14,15,16,17,18,19,20,21,22,23]. The overall screening and selection process is visually summarized in Figure 1.

### 3.2. Basic Features of Included Research

This meta-analysis encompassed 12 studies published between 2004 and 2023, collectively involving 704,568 participants. Of these, 10 studies were designed as retrospective cohort analyses, while the remaining 2 studies were retrospective case–control studies. Among the included studies, eight articles provided data on age [12,14,15,16,19,20,22,23], four articles provided data on ASA classification [14,18,20,23], seven articles provided information on bleeding disorders [12,13,17,19,20,21,23], four articles provided data on surgery type [12,13,14,18], five articles provided data on comorbid chronic diseases [19,20,21,22,23], five articles provided gender data [12,14,15,19,21], three articles provided data on infection risk information [13,17,23], and two articles provided data on anesthesia type [14,15]. These research features are provided in Table 1.

### 3.3. Quality Assessment of the Studies

Our analysis included 10 retrospective cohort studies (RCSs) [12,13,15,16,17,18,19,20,21,23] and 2 retrospective case–control studies (RCCs) [14,22]. The Newcastle–Ottawa Scale (NOS) assessed study quality across three domains: Study Design (case definition, case representativeness, selection of controls, and definition of controls; four criteria), Comparability (control for confounders; one criterion, with up to two points), and Exposure (ascertainment, method consistency, and non-response; three criteria). For Comparability, studies were awarded up to two points for adjusting for key factors like age, ASA classification, or chronic diseases. The studies scored 7–8 points, indicating that they were high quality. Two assessors independently evaluated the studies, resolving discrepancies via discussion with a third researcher. The results of the assessment are presented in Table 2.

### 3.4. Meta-Analysis

This analysis highlights the primary influential factors in this study along with their statistical results, covering odds ratios (ORs), 95% confidence intervals (CIs), *p*-values, and heterogeneity indices (I^2^s). These variables were demonstrated to be statistically significant and clinically meaningful in relation to postoperative readmission, as shown in Table 3.

**Figure 2:**
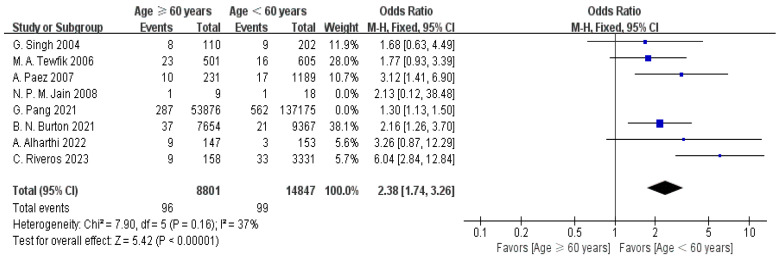
Forest plot of age (≥60 years) vs. readmission: OR = 2.38, 95% CI: 1.74–3.26, FEM, and I^2^ = 37% [12,14,15,16,19,20,22,23].

Eight articles provided age data [12,14,15,16,19,20,22,23]. The initial meta-analysis revealed substantial heterogeneity among the included studies (I^2^ = 70%). To address this, a sensitivity analysis was performed. After the exclusion of two studies, heterogeneity was notably reduced (I^2^ = 37%). Subsequently, a fixed-effect model (FEM) was applied, revealing a significantly notable age difference among the readmission and non-readmission cohorts (OR = 2.38, 95% CI: 1.74–3.26, *p* < 0.00001), as displayed in Figure 2.

**Figure 3:**
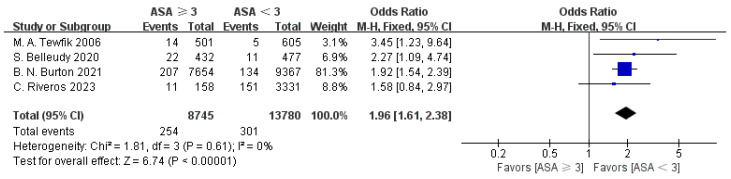
Forest plot of ASA (≥3) vs. readmission: OR = 1.96, 95% CI: 1.61–2.38, FEM, and I^2^ = 0% [14,18,20,23].

Four articles provided ASA data [14,18,20,23]. The results of the meta-analysis demonstrated low heterogeneity among these studies (I^2^ = 0%), supporting the use of a fixed-effect model (FEM). A statistically significant association was observed between ASA scores and the likelihood of hospital readmission (OR = 1.96, 95% CI: 1.61–2.38, *p* < 0.00001), as displayed in Figure 3.

**Figure 4:**
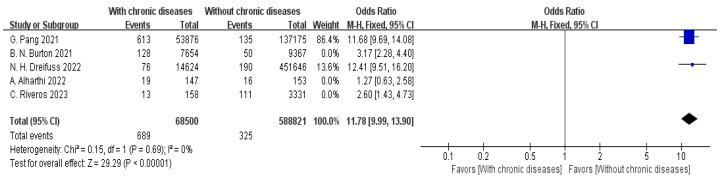
Forest plot of chronic comorbidities vs. readmission: OR = 11.78, 95% CI: 9.99–13.90, FEM, and I^2^ = 0% [19,20,21,22,23].

Five articles provided data on comorbid chronic diseases [19,20,21,22,23]. The meta-analysis showed high heterogeneity between the studies (I^2^ = 96%). To address this, a sensitivity assessment was conducted, and the three studies causing variability were removed. As a result, the heterogeneity was markedly reduced (I^2^ = 0%). Applying a fixed-effect model (FEM), a statistically significant association was observed between chronic comorbidities and hospital readmission status. Patients in the readmission group exhibited a significantly higher prevalence of chronic comorbid conditions compared to those who were not readmitted (OR = 11.78, 95% CI: 9.99–13.90, *p* < 0.00001), as displayed in Figure 4.

**Figure 5:**
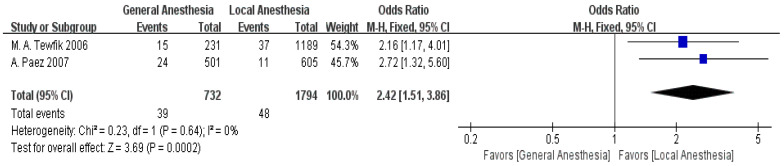
Forest plot of general anesthesia vs. readmission: OR = 2.42, 95% CI: 1.51–3.86, FEM, and I^2^ = 0% [14,15].

Two articles provided data on anesthesia type [14,15]. The meta-analysis showed low heterogeneity between the studies (I^2^ = 0%). Using the fixed-effect model (FEM), a notable difference in anesthesia types was observed between the non-readmission and readmission groups (OR = 2.42, 95% CI: 1.51–3.86, *p* = 0.0002), as displayed in Figure 5.

**Figure 6:**
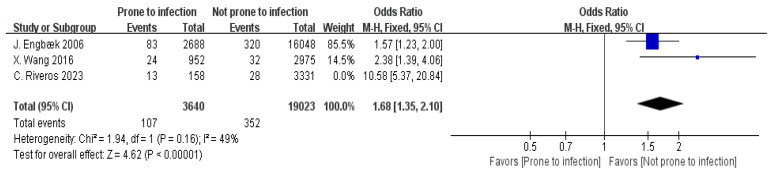
Forest plot of infection risk vs. readmission: OR = 1.68, 95% CI: 1.35–2.10, FEM, and I^2^ = 49% [13,17,23].

Three articles provided data on infection risk [13,17,23]. The analysis showed substantial variability between the studies (I^2^ = 93%). Following a sensitivity analysis that excluded one of the studies, heterogeneity was notably reduced (I^2^ = 49%). Based on the fixed-effect model (FEM), the analysis demonstrated a statistically notable difference in infection risk among the non-readmission and readmission cohorts (OR = 1.68, 95% CI: 1.35–2.10, *p* < 0.00001), as displayed in Figure 6.

**Figure 7:**
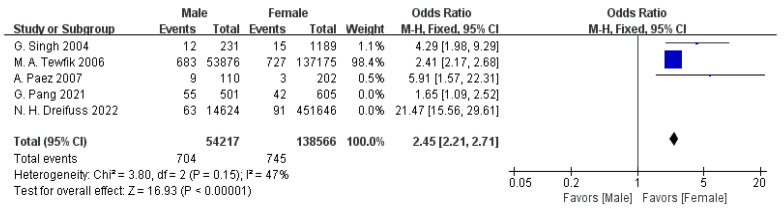
Forest plot of male gender vs. readmission: OR = 2.45, 95% CI: 2.21–2.71, FEM, and I^2^ = 47% [12,14,15,19,21].

Five articles provided data on gender [12,14,15,19,21]. The meta-analysis showed substantial heterogeneity between the studies (I^2^ = 98%). To address this, a sensitivity analysis was performed, and two studies were excluded, resulting in a marked reduction in heterogeneity (I^2^ = 47%). Following this adjustment, a fixed-effect model (FEM) was applied, which demonstrated a statistically significant association between gender and readmission status (OR = 2.45, 95% CI: 2.21–2.71, *p* < 0.00001), as displayed in Figure 7.

**Figure 8:**
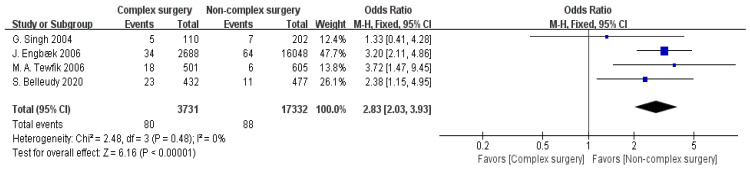
Forest plot of complex surgery vs. readmission: OR = 2.83, 95% CI: 2.03–3.93, FEM, and I^2^ = 0% [12,13,14,18].

Four articles provided data on surgery type [12,13,14,18]. The meta-analysis revealed low heterogeneity among these studies (I^2^ = 0%), justifying the application of a fixed-effect model (FEM). Based on this model, a statistically significant association was identified between surgery type and hospital readmission status (OR = 2.83, 95% CI: 2.03–3.93, *p* < 0.00001), as displayed in Figure 8.

**Figure 9:**
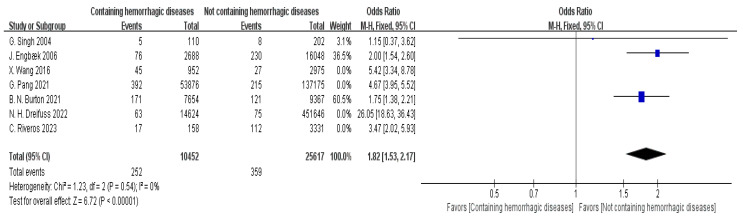
Forest plot of hemorrhagic diseases vs. readmission: OR = 1.82, 95% CI: 1.53–2.17, FEM, and I^2^ = 0% [12,13,17,19,20,21,23].

Seven articles provided data on bleeding disorders [12,13,17,19,20,21,23]. The meta-analysis indicated high heterogeneity between the studies (I^2^ = 97%). To address this, a sensitivity analysis was performed, leading to the exclusion of four studies. This adjustment markedly reduced the heterogeneity (I^2^ = 0%). Based on the fixed-effect model (FEM), a statistically significant association was observed between bleeding disorders and hospital readmission status (OR = 1.82, 95% CI: 1.53–2.17, *p* < 0.00001), as displayed in Figure 9.

In the meta-analysis, certain factors exhibited high heterogeneity, as assessed by I^2^ values exceeding 50%, reflecting variability in the study designs and populations. After conducting a sensitivity analysis to address this variability and excluding some studies, the heterogeneity was significantly reduced. A random-effects model was initially chosen to account for this anticipated variability, providing more conservative estimates. Exclusions were guided by specific issues such as divergent methodologies or incomplete data to ensure clinical relevance and reduce heterogeneity. To explain the reasons for the reduction in heterogeneity, a summary of the excluded studies, the authors, the reasons for removal, and the resulting I^2^ values for each affected risk factor is provided in Table 4.

## 4. Discussion

This meta-analysis evaluated the influence of diverse preoperative risk factors on unplanned readmissions following day surgery, with the goal of offering stronger evidence-based support for clinical decision-making and optimizing patient management strategies to minimize avoidable postoperative readmissions. Prior studies showed conflicting findings on risk factors like ASA classification, with some linking an ASA ≥ 3 to a higher readmission risk, while others found no association [7,8]. These inconsistencies arise from regional variations in healthcare practices or differences in surgical types [12,16]. Our meta-analysis resolves these by synthesizing 12 studies (704,568 patients) using sensitivity analyses to reduce heterogeneity [Figure 3] and adjusted odds ratios to control for confounders.

The widespread use of day surgery has not only effectively reduced patient hospitalization time and medical costs but also increased hospital bed turnover rates. However, the issue of postoperative readmissions continues to significantly impact patient recovery and healthcare quality [24,25]. Therefore, identifying and managing key preoperative risk factors has become a critical step in improving patient safety, enhancing surgical success rates, and reducing postoperative readmissions [26,27]. Moreover, in-depth analyses of these risk factors can help healthcare professionals make more scientific assessments before surgery and implement more targeted interventions, effectively lowering the risk of postoperative readmissions.

An age ≥ 60 years was recognized as an distinct risk factor for readmission following day surgery (OR = 2.38, 95% CI: 1.74–3.26). This finding underscores the clinical need for comprehensive preoperative assessments in elderly patients to address their elevated risk of complications, which drives readmissions [28,29,30]. This result aligns with many previous studies, as older patients often experience slower postoperative recovery due to decreased physical function, increased chronic diseases, and reduced metabolic capacity [28]. As patients age, their immune system gradually weakens, and their physiological recovery ability significantly diminishes, resulting in generally higher postoperative complication rates. Elderly patients often have multiple chronic conditions, such as hypertension, diabetes, and osteoporosis. These comorbidities not only are associated with an increased risk of postoperative complications but also significantly raise the probability of readmission after surgery [29,30]. Moreover, older patients may experience a decline in physical function, including weakened muscle strength and poor balance, making the postoperative rehabilitation process more challenging [31]. Therefore, preoperative assessments of elderly patients must be more detailed and comprehensive. They should not only evaluate their basic health status but also consider factors such as functional status, chronic disease management, medication use, and activities of daily living, in order to implement appropriate interventions [32,33]. Such assessments enable personalized postoperative plans, including physical therapy and nutritional support, to enhance recovery and reduce readmissions [34,35]. Additionally, an early, personalized rehabilitation plan should be initiated postoperatively, including physical therapy, nutritional support, and psychological interventions, to help elderly patients recover more quickly and regain their daily living abilities as soon as possible, reducing readmission rates and improving their overall quality of life [34,35].

Patients classified as ASA ≥ 3 (OR = 1.96, 95% CI: 1.61–2.38) and those with comorbid chronic diseases (OR = 11.78, 95% CI: 9.99–13.90) are at a greater risk of postoperative readmission. A higher ASA score typically indicates poorer overall health, which complicates postoperative recovery [36]. The high OR for chronic diseases highlights their critical clinical impact, as conditions like diabetes, cardiovascular diseases, and chronic kidney disease are associated with impaired wound healing and immune function, elevating the risk of infections or acute exacerbations requiring readmission [37,38,39]. The high OR (11.78) partly reflects unmeasured severity, as the analysis did not distinguish between mild and severe chronic conditions, introducing confounding that overestimates the association. This clinical impact underscores the importance of preoperative optimization, such as stabilizing blood glucose or optimizing cardiac function, to improve outcomes and reduce readmission burdens. Given that chronic conditions such as diabetes often contribute to higher ASA scores, there is an overlap between these factors, introducing collinearity that could affect the independence of their effects. While adjusted odds ratios were used to control for confounders, the limited data across studies precluded a full assessment of this collinearity, suggesting a need for future research to clarify their distinct contributions to readmission risk. Chronic conditions, including diabetes, cardiovascular diseases, and chronic kidney disease are associated with impaired wound healing, postoperative infections, and other complications. Diabetic patients typically have weaker immune systems, and due to prolonged blood glucose fluctuations, they have delayed wound healing. For diabetic patients, prioritizing blood glucose control before surgery can mitigate postoperative complications [40]. In patients suffering from cardiovascular diseases, particularly those diagnosed with coronary heart disease or heart failure, postoperative recovery heart failure is a risk [38]. Patients with chronic kidney disease are at a higher risk of kidney function deterioration after surgery. If nephrotoxic drugs are used during surgery, the risk of postoperative complications with kidney function increases [39]. Therefore, for these high-risk patients, clinicians should conduct a comprehensive medical evaluation before surgery, with a focus on controlling and optimizing underlying conditions. For patients with hypertension, antihypertensive medications should be adjusted based on the patient’s condition to ensure their blood pressure is within an ideal range, thereby decreasing the risk of cardiovascular events postoperatively. For patients with cardiovascular diseases, detailed cardiac function assessments, including electrocardiograms, echocardiograms, and other tests, should be performed to evaluate cardiac reserves. If necessary, further cardiac treatment should be provided to reduce the incidence of postoperative cardiac events. Additionally, postoperative monitoring of vital signs should be strengthened, with close observation of important indicators such as blood glucose, blood pressure, cardiac function, and renal function, with timely adjustments and interventions. Strict measures for preventing and managing complications should be implemented to prevent postoperative complications and promote a smooth recovery for these patients [40].

General anesthesia is closely linked to the risk of postoperative readmission (OR = 2.42, 95% CI: 1.51–3.86). This finding suggests that general anesthesia is associated with increased complication risks, such as respiratory or cognitive issues, suggesting the need for careful consideration of anesthesia types to minimize readmissions [41,42,43,44]. However, findings from prior randomized controlled trials (RCTs) [10], indicate no significant difference in readmission rates between general and local anesthesia in minor surgeries, which may reflect context-specific factors influencing our results. This result suggests that general anesthesia is associated with an impact on postoperative recovery, particularly by being linked to extended recovery time and an increased the risk of complications. General anesthesia is associated with a longer period of wake-up recovery but is linked to short-term instability in the respiratory and circulatory systems, thus increasing the risk of postoperative complications [41]. During general anesthesia, the depressant effect of anesthetic drugs on the central nervous system is associated with respiratory depression, cardiovascular instability, and other issues, especially in patients with multiple underlying conditions, where such instability is more pronounced [42]. The recovery period after anesthesia is typically longer, with patients reporting symptoms such as nausea, vomiting, and breathing difficulties, which affect the postoperative recovery process and are associated with a lengthened hospital stay [43]. Additionally, general anesthesia is associated with postoperative cognitive dysfunction (such as postoperative delirium), particularly in elderly patients, affecting their cognitive recovery and overall quality of life [44]. In contrast, local or regional anesthesia is associated with fewer complications and shorter recovery times in many day surgeries [45]. Local and regional anesthesia is able to precisely anesthetize the surgical area without affecting the overall physiological state, reducing interference with the patient’s general physiology, thus shortening the wake-up time and reducing the incidence of postoperative complications [46]. Another advantage of local anesthesia is that it is associated with minimal impacts on cognitive function during recovery, which help improve the quality of recovery [47]. Therefore, during preoperative evaluations, clinicians should prioritize local or regional anesthesia based on the patient’s health status, surgery type, and individual differences, reducing the use of general anesthesia in low-risk day surgeries [48]. Preferring local anesthesia where feasible reduces complications, particularly in high-risk groups [49]. For patients requiring general anesthesia, the preoperative assessment should focus on evaluating the patient’s cardiopulmonary function, medication history, and previous anesthesia history to minimize the impact of anesthesia on the patient’s body [49]. Additionally, postoperative care should emphasize close monitoring and support for patients undergoing general anesthesia to ensure a smooth transition to the recovery phase, facilitate early recovery, and reduce the risk of readmission.

Gender significantly influences postoperative readmission risk, with male patients showing a higher likelihood of readmission (OR = 2.45, 95% CI: 2.21–2.71, *p* < 0.00001). This finding highlights the need for gender-sensitive interventions, such as enhanced health education for males, to improve adherence and reduce readmissions [50,51]. In contrast, a meta-analysis reported no significant gender difference, possibly due to variations in population demographics or behavioral adjustments [7]. This association stems from biological and behavioral factors. Biologically, differences in hormonal profiles or immune responses are associated with differences in pain perception and recovery rates, which contribute to higher complication rates in males [50]. Behaviorally, males often exhibit lower adherence to postoperative care plans, such as physical therapy or medication regimens, which are associated with a delayed recovery [51]. Cultural biases further exacerbate these risks: societal expectations of male stoicism are associated with the underreporting of pain, hindering timely pain management, while females are generally more likely to seek medical assistance promptly [52,53]. Clinicians should adopt gender-sensitive approaches, including tailored pain assessments and enhanced health education for male patients, to improve adherence and reduce readmission risks [54,55,56].

The complexity of the surgical procedure significantly influences the risk of postoperative readmission (OR = 2.83, 95% CI: 2.03–3.93). Clinically, this advocates for prioritizing minimally invasive techniques to reduce complication rates and readmissions [57,58,59]. Complex surgeries are associated with longer operation times, higher complication rates, and a more challenging postoperative recovery, which indicates that patients face additional challenges in the recovery process. For example, open surgery is associated with larger incisions and longer postoperative care, with greater intraoperative blood loss, which is linked to disruption of the patient’s immune system, leading to delayed recovery and a higher risk of postoperative infections. In contrast, minimally invasive surgeries, completed through smaller incisions and with shorter operation times, are associated with reduced physiological interference, thereby shortening the recovery time and lowering complication rates [57]. The advantage of minimally invasive surgery lies in its faster recovery, allowing patients to resume normal activities earlier, which reduces the risk of readmission. For patients undergoing complex surgeries, a detailed risk assessment and thorough preoperative screening of the patient’s baseline health status, medical history, and comorbidities that may impact the surgery is essential [58]. Preoperative optimization measures, such as controlling blood glucose levels, adjusting antihypertensive medications, and assessing cardiopulmonary function, reduce the occurrence of intraoperative and postoperative complications. While minimally invasive surgery offers clear advantages in many cases, it is not suitable for all patients, and personalized assessments and decisions are necessary [59]. For patients with complex conditions, traditional open surgery may still be the inevitable choice. Therefore, the clinical team should prioritize postoperative care, ensuring that patients receive timely interventions and support after surgery to minimize the risk of complications and enhance recovery quality.

Patients with bleeding disorders have an elevated risk of postoperative readmission (OR = 1.82, 95% CI: 1.53–2.17). This emphasizes the clinical importance of preoperative hematologic assessments to prevent complications like hematomas that lead to readmissions [60,61,62,63]. Individuals with bleeding disorders typically have abnormalities in their coagulation mechanisms, which are associated with increased difficulty controlling bleeding postoperatively. This, in turn, is associated with an increased recovery time and raises the risk of readmission [60]. Patients with hemophilia or other platelet dysfunctions are prone to significant bleeding during and after surgery, which is associated with complications in the recovery process and is linked to an increased risk of the risk of postoperative infections, hematomas, and other complications [61]. Therefore, preoperative evaluations are particularly important for patients with bleeding disorders. Clinicians should conduct a detailed hematologic assessment, specifically evaluating coagulation function, platelet function, international normalized ratio (INR), and prothrombin time (PT) [62]. These assessments help healthcare providers identify potential bleeding risks and guide intraoperative and postoperative management. During surgery, surgeons should pay particular attention to bleeding control, using hemostatic measures such as electrocautery, tourniquets, or local hemostatic agents when necessary. Moreover, postoperative recovery for patients with bleeding disorders requires more intensive monitoring, particularly of platelet counts and coagulation factor levels, to ensure that bleeding is effectively controlled [63]. In the postoperative period, nursing care for these patients should be more meticulous. In addition to routine vital sign monitoring, healthcare providers should remain highly vigilant for signs of bleeding, such as wound seepage or hematoma formation. Furthermore, the rehabilitation period for these patients is typically longer. When providing discharge instructions to patients and their families, healthcare providers should emphasize the risk of postoperative bleeding and ensure that the patient strictly follows the postoperative care plan to minimize the risk of readmission due to improper medication use or neglect of care.

Patients with a tendency to have infections have a significantly elevated risk of postoperative readmission (OR = 1.68, 95% CI: 1.35–2.10). This finding stresses the need for preoperative infection risk evaluations and antibiotic prophylaxis to prevent surgical site infections that lead to readmissions [64,65,66,67]. These patients are associated with higher infection risks postoperatively, which lead to a delayed recovery, increased complication rates, and even the need for readmission for further treatment. Infections directly affect the patient’s immune function, increasing the risk of both surgical site and systemic infections, which are further associated with prolonged hospital stays and increase the likelihood of readmission [64]. For patients with infection risks, preoperative assessment is crucial. A comprehensive evaluation of the patient’s immune system function and identification of any potential sources of infection should be performed [65]. Proactive infection prevention measures should be taken before surgery, including the appropriate use of antibiotics for infection prophylaxis and ensuring sterile techniques are followed during the procedure to reduce the risk of postoperative infections [66]. During postoperative recovery, patients with infection tendencies need to be closely monitored for indicators including core body heat, leukocyte numbers, and concentrations of C-reactive protein (CRP). The early identification of infections allows for timely intervention [67]. Healthcare providers should initiate appropriate antimicrobial therapy at the earliest signs of infection, develop individualized prevention and treatment strategies, and enhance postoperative recovery quality. This approach will help reduce complications and lower the risk of readmission.

### Limitations of the Study

This study’s findings are tempered by retrospective bias in the included studies and heterogeneity across patient populations and methodologies. A major limitation of this study is that many of the included studies were retrospective, which often introduces a certain degree of retrospective bias. This bias stems from selective recording of data and the inability to control for potential confounders in retrospective analyses, potentially inflating odds ratios (ORs) by up to 10–20% in similar meta-analyses, though the exact impact in our study requires further prospective validation, which may affect the accuracy and generalizability of the results. Retrospective studies cannot completely eliminate selection bias and systematic errors in their research design, which could result in high-risk patients not being included in the study in a timely manner, or certain data not being fully recorded. This may limit the ability to establish definitive causal relationships between preoperative risk factors and unplanned readmissions. To address this, we employed adjusted odds ratios (ORs) from multivariable regression models to account for confounders such as age, comorbidities, and surgical characteristics, and conducted sensitivity analyses to enhance the reliability of our findings. This, in turn, could impact the assessment of preoperative risk factors and their effects. Future studies should adopt a prospective design, which allows for data collection under stricter control conditions, enabling further validation of the applicability and accuracy of these preoperative risk factors across different populations. Additionally, the high heterogeneity among the studies included in this research is another limitation. The diversity of patient populations, variations in surgery types, and differences in anesthesia methods may contribute to this heterogeneity. For example, heterogeneity (I^2^ > 50%), such as for gender (I^2^ = 98% initially), potentially overestimated ORs by approximately 5–15%, as indicated by sensitivity analyses reducing the I^2^ values, though the precise effect varies by factor. To mitigate this, we conducted sensitivity analyses, excluding studies contributing significantly to variability, to enhance the robustness and consistency of our results. Furthermore, the analysis of general versus local/regional anesthesia was constrained by data from only two studies [14,15], which may temper the robustness of our conclusions regarding its impact on readmission risk. The manifestation and impact of preoperative risk factors can vary among different patient groups, making the synthesis of results more complex. Future research should focus on reducing heterogeneity between studies by collecting more diverse and balanced patient data under a multi-center collaboration, aiming to minimize the impact of differences in sample sources, study designs, and methodologies. Although this study provides valuable evidence supporting the evaluation of preoperative risk factors, further validation of the robustness and generalizability of these risk factors is needed through prospective, multi-center, large-sample studies. Therefore, future research should aim to improve and refine preoperative risk assessment models based on multi-dimensional and multi-level approaches, exploring personalized management plans suitable for different patient groups and types of surgeries, in order to achieve higher levels of patient safety and better surgical outcomes.

## 5. Conclusions

This meta-analysis underscores the critical role of preoperative risk factors in influencing unplanned readmissions after day surgery, emphasizing the need for a tailored approach to patient management. The findings highlight the importance of enhanced preoperative assessments to identify high-risk patients, enabling targeted interventions such as preoperative optimization for patients with an ASA ≥ 3 with stabilizing chronic conditions, preferring local anesthesia where appropriate, and conducting hematologic assessments for bleeding disorders to mitigate readmission risks, as supported by prior studies [15,23]. Although the results are robust, they are limited by the retrospective nature of the included studies and study heterogeneity. Therefore, prospective multi-center studies are needed to validate these findings and to refine personalized management strategies for diverse patient populations.

## Figures and Tables

**Table 1:** The fundamental features of the selected studies.

Author	Nation	Research Design	Type of Surgery	Research Timeline	Data Source	Sample Size	Outcomes
G. Singh 2004 [12]	UK	RCS	Septoplasty, Lesion Excision, Polypectomy, Sinus Surgery	January 2002–June 2003	SC	312	①⑥⑦⑧
J. Engbæk 2006 [13]	DNK	RCS	Hernia Repair, Knee Arthroscopy, Surgical Abortion	January 1996–December 2000	MC	18,736	⑤⑦⑧
M. A. Tewfik 2006 [14]	CAN	RCC	Open Neck Biopsy, Functional Endoscopic Sinus Surgery (FESS), Tonsillectomy, Mastoidectomy	July 2000–July 2004	SC	1106	①②④⑥⑦
A. Paez 2007 [15]	ESP	RCS	Scrotal Surgery, Penile Surgery, Bladder Surgery, Ureteroscopy	16 months	SC	1420	①④⑥
N. P. M. Jain 2008 [16]	UK	RCS	Arthroscopic Acromioplasty (ASAD)	June 2002–June 2004	SC	27	①
X. Wang 2016 [17]	CHN	RCS	Cholangioscopy Stone Removal, Colon Polypectomy, Laparoscopic Cholecystectomy (LC), Endoscopic Nasal Surgery	February–September 2014	SC	3927	⑤⑧
S. Belleudy 2020 [18]	FRA	RCS	Functional Septoplasty, Ethmoidectomy, Middle Meatal Antrostomy, Frontal Sinusectomy, Endoscopic Sphenoid Sinusectomy	2016–2017	SC	909	②⑦
G. Pang 2021 [19]	CAN	RCS	Appendectomy, Mastectomy, Cholecystectomy, Hernia Repair	2005–2017	MC	191,051	①③⑥⑧
B. N. Burton 2021 [20]	USA	RCS	Total Shoulder Arthroplasty (TSA), Hemiarthroplasty	2012–2016	MC	17,021	①②③⑧
N. H. Dreifuss 2022 [21]	USA	RCS	Laparoscopic Sleeve Gastrectomy (SG)	2015–2018	MC	466,270	③⑥⑧
A. Alharthi 2022 [22]	SAU	RCC	Otorhinolaryngology (ENT) Surgery, General Surgery, Urological Surgery, Orthopedic Surgery	2009–2021	SC	300	①③
C. Riveros 2023 [23]	USA	RCS	Holmium Laser Enucleation of The Prostate (HoLEP)	2011–2019	MC	3489	①②③⑤⑧

USA—United States; UK—United Kingdom; CAN—Canada; FRA—France; DNK—Denmark; ESP—Spain; CHN—China; SAU—Saudi Arabia; RCS—Retrospective cohort study; RCC—Retrospective case–control study; SC—Single-center; MC—Multi-center. ① Patient age, ② ASA physical status, ③ Chronic comorbidities, ④ Anesthesia type, ⑤ Infection risk, ⑥ Gender, ⑦ Surgery type, and ⑧ Bleeding disorders.

**Table 2:** NOS scores.

Study	Study Design				Comparability	Exposure			Scores
	Case Definition	Case Representativeness	Selection of Controls	Definition of Controls	Comparability of Cases and Controls	Ascertainment of Exposure	Same Methods of Ascertainment for Cases and Controls	Non-/Response Rate	
G. Singh 2004 [12]	★	★	★	★	★	★	★	★	8 stars
J. Engbæk 2006 [13]	★	★	★	★	★	★	★	★	8 stars
M. A. Tewfik 2006 [14]	★	★	★	★	★	★	★	★	8 stars
A. Paez 2007 [15]	★	★	★	★	★	★		★	7 stars
N. P. M. Jain 2008 [16]	★	★	★	★	★	★	★		7 stars
X. Wang 2016 [17]	★	★	★	★	★	★	★		7 stars
S. Belleudy 2020 [18]	★	★	★	★	★	★	★	★	8 stars
G. Pang 2021 [19]	★	★	★	★	★	★	★	★	8 stars
B. N. Burton 2021 [20]	★	★	★	★	★	★	★	★	8 stars
N. H. Dreifuss 2022 [21]	★	★	★	★	★	★	★	★	8 stars
A. Alharthi 2022 [22]	★	★	★	★	★	★	★		7 stars
C. Riveros 2023 [23]	★	★	★	★	★	★	★	★	8 stars

High-quality literature rated 7–8 stars.

**Table 3:** Significant risk factors.

Significant Risk Factors	OR	95% CI	*p*-Value	I^2^
Chronic Comorbidities	OR = 11.78	9.99–13.90	<0.00001	96% → 0%
Surgery Type	OR = 2.83	2.03–3.93	<0.00001	0%
Gender (Male)	OR = 2.45	2.21–2.71	<0.00001	98% → 47%
Age	OR = 2.38	1.74–3.26	<0.00001	70% → 37%

The arrows demonstrate that the sensitivity analysis led to a significant reduction in heterogeneity, emphasizing the impact of the excluded studies and consequently strengthening the robustness and credibility of the overall findings.

**Table 4:** Excluded studies and their influence.

Risk Factor	Excluded Studies (Author, Year)	Reason for Exclusion	I^2^
Age	N. P. M. Jain 2008 [16], G. Pang 2021 [19]	Age classification diverges from other research [16]; Statistical models or methods differ from those used in other studies [19]	70% → 37%
Chronic Comorbidities	B. N. Burton 2021 [20], A. Alharthi 2022 [22], C. Riveros 2023 [23]	No complete categorized data on chronic comorbidities provided [20]; Classification of chronic comorbidities differs from other studies [22]; Inclusion criteria differ significantly from other studies, with different patient populations [23]	96% → 0%
Gender	G. Pang 2021 [19], N. H. Dreifuss 2022 [21]	Statistical models or methods differ from those used in other studies [19]; Key variable definitions differ from those in other studies [21]	98% → 47%
Infection Risk	C. Riveros 2023 [23]	Inclusion criteria differ significantly from other studies, with different patient populations	93% → 49%
Bleeding Disorders	X. Wang 2016 [17], G. Pang 2021 [19], N. H. Dreifuss 2022 [21], C. Riveros 2023 [23]	The objectives of the study do not correspond with the focus of the research [17]; Statistical models or methods differ from those used in other studies [19]; Key variable definitions differ from those in other studies [21]; Inclusion criteria differ significantly from other studies, with different patient populations [23]	97% → 0%

## Data Availability

The data presented in this study are available on request from the corresponding author due to privacy.

## Abbreviations

OR	Odds Ratio
CI	Confidence Interval
*p*	Significance Probability

## References

[1] Zhang H., Gao X., Chen Z. (2025). The Impact of Preoperative Risk Factors on Delayed Discharge in Day Surgery: A Meta-Analysis. Healthcare.

[2] Zeng Y., Wen J., Zhan S., Hao X., Wang Y., Zhang L. (2025). Cost-Effective Day Surgery for Arteriovenous Fistula Stenosis: A Viable Model for Hemodialysis Patients. Med. Sci. Monit..

[3] Herbst M.O., Price M.D., Soto R.G. (2017). Pain related readmissions/revisits following same-day surgery: Have they decreased over a decade?. J. Clin. Anesth..

[4] Seleem M.I., Gerges S.S., Shreif K.S., Ahmed A.E., Ragab A. (2011). Laparoscopic cholecystectomy as a day surgery procedure: Is it safe?--an egyptian experience. Saudi J. Gastroenterol..

[5] Pansard E., Klouche S., Bauer T., Ménigaux C., Hardy P., Meziane A.M. (2020). Can primary total hip arthroplasty be performed in an outpatient setting? Prospective feasibility and safety study in 321 patients in a day-surgery unit. Orthop. Traumatol. Surg. Res..

[6] Serra-Aracil X., Labró-Ciurans M., Rebasa P., Mora-López L., Pallisera-Lloveras A., Serra-Pla S., Gracia-Roman R., Navarro-Soto S. (2019). Morbidity after transanal endoscopic microsurgery: Risk factors for postoperative complications and the design of a 1-day surgery program. Surg. Endosc..

[7] Lalonde S., Wood G.C. (2019). Short stay total joint arthroplasty program: Patient factors predicting readmission. Can. J. Surg..

[8] Sathiyakumar V., Molina C.S., Thakore R.V., Obremskey W.T., Sethi M.K. (2015). ASA score as a predictor of 30-day perioperative readmission in patients with orthopaedic trauma injuries: An NSQIP analysis. J. Orthop. Trauma.

[9] Fosnot C.D., Fleisher L.A., Keogh J. (2015). Providing value in ambulatory anesthesia. Curr. Opin. Anaesthesiol..

[10] Beaulieu R.J., Locham S., Nejim B., Dakour-Aridi H., Woo K., Malas M.B. (2019). General anesthesia is associated with reduced early failure among patients undergoing hemodialysis access. J. Vasc. Surg..

[11] Mo K., Gupta A., Al Farii H., Raad M., Musharbash F., Tran B., Zheng M., Lee S.H. (2022). 30-day postoperative sepsis risk factors following laminectomy for intradural extramedullary tumors. J. Spine Surg..

[12] Singh G., McCormack D., Roberts D.R. (2004). Readmission and overstay after day case nasal surgery. BMC Ear Nose Throat Disord..

[13] Engbaek J., Bartholdy J., Hjortsø N.C. (2006). Return hospital visits and morbidity within 60 days after day surgery: A retrospective study of 18,736 day surgical procedures. Acta Anaesthesiol. Scand..

[14] Tewfik M.A., Frenkiel S., Gasparrini R., Zeitouni A., Daniel S.J., Dolev Y., Kost K., Samaha M., Sweet R., Tewfik T.L. (2006). Factors affecting unanticipated hospital admission following otolaryngologic day surgery. J. Otolaryngol..

[15] Paez A., Redondo E., Linares A., Rios E., Vallejo J., Sanchez-Castilla M. (2007). Adverse events and readmissions after day-case urological surgery. Int. Braz. J. Urol..

[16] Jain N.P., Ogonda L., Trimmings N.P. (2008). Age as a predictive factor for in-patient admission following day-case shoulder arthroscopic sub-acromial decompression—A district general hospital audit. Ann. R. Coll. Surg. Engl..

[17] Wang X., Ma H., Li Z. (2016). Analysis of the Cause of the Postoperative Unplanned Readmission of Ambulatory Surgery Patients. Sichuan Med. J..

[18] Belleudy S., Kérimian M., Legrenzi P., Alharbi A., de Gabory L. (2021). Assessment of quality and safety in rhinologic day surgery. Eur. Ann. Otorhinolaryngol. Head. Neck Dis..

[19] Pang G., Kwong M., Schlachta C.M., Alkhamesi N.A., Hawel J.D., Elnahas A.I. (2021). Safety of Same-day Discharge in High-risk Patients Undergoing Ambulatory General Surgery. J. Surg. Res..

[20] Burton B.N., Finneran J.J., Angerstein A., Ross E., Mitchell A., Waterman R.S., Elsharydah A., Said E.T., Gabriel R.A. (2021). Demographic and clinical factors associated with same-day discharge and unplanned readmission following shoulder arthroplasty: A retrospective cohort study. Korean J. Anesthesiol..

[21] Dreifuss N.H., Xie J., Schlottmann F., Cubisino A., Baz C., Vanetta C., Mangano A., Bianco F.M., Gangemi A., Masrur M.A. (2022). Risk Factors for Readmission After Same-Day Discharge Sleeve Gastrectomy: A Metabolic and Bariatric Surgery Accreditation and Quality Improvement Program Database Analysis. Obes. Surg..

[22] Alharthi A.A., Mohammed A., Jamil M., Mehboob A., Huda A.U. (2022). Evaluation of risk factors for unanticipated hospital admission following ambulatory surgery—An observational study. Saudi J. Anaesth..

[23] Riveros C., Di Valerio E., Bacchus M., Chalfant V., Leelani N., Thomas D., Jazayeri S.B., Costa J. (2023). Predictors of readmission and impact of same-day discharge in holmium laser enucleation of the prostate. Prostate Int..

[24] Almashari Y.M., Allarakia Y., Barradah O., Alqozi Y., Almoraei A.M., Alshaya R.A., Muawad R. (2024). Incidence of Unplanned Readmissions After One-Day Surgery Discharge Among Pediatric Patients: A Retrospective Study From a Tertiary Care Hospital in Saudi Arabia. Cureus.

[25] Kremel D., Siatos D., Jaafari F.A. (2020). Ureteroscopy in the day case setting: It’s worth it! Retrospective single surgeon outcomes analysis during service relocation (inpatient to daycase) in a DGH. J. Clin. Urol..

[26] Soliman M.F.A. (2024). Predictors of Postoperative 30 Days Unplanned Readmission among Patients Undergoing Cardiac Surgeries. Assiut Sci. Nurs. J..

[27] Johns W.L., Layon D., Golladay G.J., Kates S.L., Scott M., Patel N.K. (2020). Preoperative Risk Factor Screening Protocols in Total Joint Arthroplasty: A Systematic Review. J. Arthroplast..

[28] Winkelman W.D., Jaresova A., Modest A.M., Richardson M.L. (2021). Postoperative Admission, Readmission, and Complications for Patients 60 Years and Older Who Are Undergoing an Isolated Sling Procedure for Stress Incontinence: A Database Study. Female Pelvic Med. Reconstr. Surg..

[29] Li Y., Wang C., Peng M. (2021). Aging Immune System and Its Correlation With Liability to Severe Lung Complications. Front. Public Health.

[30] Yu W., Yu Y., Sun S., Lu C., Zhai J., Lei Y., Bai F., Wang R., Chen J. (2024). Immune Alterations with Aging: Mechanisms and Intervention Strategies. Nutrients.

[31] Adliah F., Rini I., Natsir W.S., Sari T. (2023). Effects of Balance and Strength Tele-Exercise (BAST) on Muscle Strength and Functional Mobility in Older Adults. J. Ilm. Kesehat. Sandi Husada.

[32] Roh Y. (2023). Comprehensive geriatric assessment for the evaluation of the health statuses of elderly patients. J. Korean Med. Assoc..

[33] Wróblewska Z., Chmielewski J.P., Florek-Łuszczki M., Nowak-Starz G., Wojciechowska M., Wróblewska I.M. (2023). Assessment of functional capacity of the elderly. Ann. Agric. Environ. Med..

[34] Chen X.W., Guo X.C., Cheng F. (2024). Impact of nutritional support on immunity, nutrition, inflammation, and outcomes in elderly gastric cancer patients after surgery. World J. Gastrointest. Surg..

[35] Munk T., Svendsen J.A., Knudsen A.W., Østergaard T.B., Thomsen T., Olesen S.S., Rasmussen H.H., Beck A.M. (2021). A multimodal nutritional intervention after discharge improves quality of life and physical function in older patients—A randomized controlled trial. Clin. Nutr..

[36] Zeidan M., Goz V., Lakomkin N., Spina N., Brodke D., Spiker W. (2020). Predictors of Readmission and Prolonged Length of Stay after Cervical Disc Arthroplasty. Spine.

[37] Swoboda L., Held J. (2022). Impaired wound healing in diabetes. J. Wound Care.

[38] Guo Z., Liu J., Sun G.-L., He Y., Song F., Chen S., Lei L., Liu B., Liu L., Chen G. (2020). Postoperative acute heart failure is an independent predictor of long-term mortality in patients with chronic kidney disease and coronary artery disease undergoing percutaneous coronary intervention. J. Am. Coll. Cardiol..

[39] Yang X., Zhang T., Zhou H.-B., Ni Z., Wang Q., Wu J., Chen Q., Qiu M.-Y., Wang Y., Fu T.-W. (2022). Acute kidney injury as an independent predicting factor for stage 3 or higher chronic kidney disease after nephrectomy. Urol. Oncol..

[40] Verrillo S., Cvach M., Hudson K., Winters B. (2019). Using Continuous Vital Sign Monitoring to Detect Early Deterioration in Adult Postoperative Inpatients. J. Nurs. Care Qual..

[41] Nabi M.-U., Anwar A.H.M.M., Ahmed T.U., Zaman M.S., Ara I., Begum M. (2024). Anesthesia-related Complications in General Surgery: Strategies for Prevention and Management. Sch. J. Appl. Med. Sci..

[42] Cardinale J., Gillespie N., Germond L. (2019). Complications of General Anesthesia. Catastrophic Perioper. Complicat. Manag..

[43] Patti C.A.M., Vieira J., Benseñor F. (2008). Incidence and prophylaxis of nausea and vomiting in post-anesthetic recovery in a tertiary teaching hospital. Rev. Bras. Anestesiol..

[44] Ehsani R., Motlagh S.D., Zaman B., Kashani S.S., Ghodraty M. (2020). Effect of General Versus Spinal Anesthesia on Postoperative Delirium and Early Cognitive Dysfunction in Elderly Patients. Anesthesiol. Pain Med..

[45] Graff V., Gabutti L., Treglia G., Pascale M., Anselmi L., Cafarotti S., La Regina D., Mongelli F., Saporito A. (2021). Perioperative costs of local or regional anesthesia versus general anesthesia in the outpatient setting: A systematic review of recent literature. Braz. J. Anesthesiol..

[46] Vacchiano M., Haupt E., Aziz K. (2023). Foot and Ankle Surgery Postoperative Complications with Regional vs General Anesthesia. Foot Ankle Orthop..

[47] Edipoglu I., Çelik F. (2019). The Associations Between Cognitive Dysfunction, Stress Biomarkers, and Administered Anesthesia Type in Total Knee Arthroplasties: Prospective, Randomized Trial. Pain Physician.

[48] McLennan L., Haines M., Graham D., Sullivan T., Lawson R., Sivakumar B. (2023). Regional Anesthesia in Upper-Limb Surgery. Ann. Plast. Surg..

[49] Kim M.K., Kim J.S., Kang H. (2025). Regional anesthesia for rapid recovery after orthopedic surgery. J. Korean Med. Assoc..

[50] Jonnavithula N., Garg H., Allenki P., Aavula K. (2021). Influence of gender on postoperative pain in percutaneous nephrolithotomy: A prospective observational study. J. Anaesthesiol. Clin. Pharmacol..

[51] Maclean M., Charest-Morin R., Stratton A., Singh S., Kelly A., Pickett G., Glennie A., Bailey C., Weber M., Attabib N. (2024). Gender differences in spine surgery for degenerative lumbar disease: Prospective cohort study. J. Neurosurg. Spine.

[52] Chou P., Lin P. (2019). ESRA19-0361 Gender difference in cancer patients’ hesitancy of analgesics use, adherence, and the effectiveness of pain management. Reg. Anesth. Pain Med..

[53] Filipescu D., Stefan M. (2020). Sex and gender differences in anesthesia: Relevant also for perioperative safety?. Best Pract. Res. Clin. Anaesthesiol..

[54] Dongre N., Dudhgaonkar S., Jaiswal K., Jaiswal N., Vaishnao L. (2020). Assessment of Utilization and Rationality of Analgesic Drugs in Perioperative Setting in a Tertiary Care Teaching Institute. J. Pharmacol. Pharmacother..

[55] Wong C.R.J., Sulaiman O.B., Tang C.S., Lim Y.H., Tan Z.T., Halim M.S.B., Leo S.W. (2024). Multimodal analgesia as part of enhanced recovery after surgery in colorectal surgery. Malays. J. Anaesthesiol..

[56] Stenberg G., Enthoven P., Molander P., Gerdle B., Stålnacke B. (2020). Patients selected to participate in multimodal pain rehabilitation programmes in primary care−a multivariate cross-sectional study focusing on gender and sick leave. Scand. J. Pain.

[57] Yolcu Y., Helal A., Alexander A., Bhatti A., Alvi M., Abode-Iyamah K., Bydon M. (2020). Minimally Invasive versus Open Surgery for Degenerative Spine Disorders for Elderly Patients: Experiences from a Single Institution. World Neurosurg..

[58] Lukas V., Dutta R., Hemal A., Tsivian M., Craven T., Deebel N., Thiel D., Pathak R. (2022). Impact of pre- and peri-operative risk factors on length of stay and hospital readmission following minimally-invasive partial nephrectomy. Asian J. Urol..

[59] Bastawrous A., Landmann R., Liu Y., Liu E., Cleary R. (2019). Incidence, associated risk factors, and impact of conversion to laparotomy in elective minimally invasive sigmoidectomy for diverticular disease. Surg. Endosc..

[60] Mitchell D., McGuire L., Khalid S., Alaraj A. (2024). Assessing the efficacy of VerifyNow platelet-function testing in predicting postoperative hemorrhagic complications of neuroendovascular surgery: A systematic review and meta-analysis (part 2). Interv. Neuroradiol. J. Peritherapeutic Neuroradiol. Surg. Proced. Relat. Neurosci..

[61] Wang K., Liu Q., Wu J., Cao Y., Wang S. (2020). The role of monitoring platelet function perioperatively and platelet transfusion for operated spontaneous intracerebral hemorrhage patients with long-term oral antiplatelet therapy: A case report. Int. J. Surg. Case Rep..

[62] Ahmed A. (2020). Monitoring of blood clotting during bleeding. Infus. Chemother..

[63] Fenger-Eriksen C. (2020). Pathophysiology of coagulation during bleeding. Infus. Chemother..

[64] Yin J., Mao W., Xiao X., Yu X., Li B.-Q., Chen F., Lin J., Zhou J., Zhou J., Tong Z. (2021). Immune Dysfunction is Associated with Readmission in Survivors of Sepsis Following Infected Pancreatic Necrosis. J. Inflamm. Res..

[65] Verdonk F., Einhaus J., Tsai A., Hédou J., Choisy B., Gaudillière D., Kin C., Aghaeepour N., Angst M., Gaudilliere B. (2021). Measuring the human immune response to surgery: Multiomics for the prediction of postoperative outcomes. Curr. Opin. Crit. Care.

[66] Bingqiang, Chen S., Jiang Z., Wu B., He Y., Wang X.-X., Li Y., Gao P., Yang X.-J. (2020). Effect of postoperative early enteral nutrition on clinical outcomes and immune function of cholangiocarcinoma patients with malignant obstructive jaundice. World J. Gastroenterol..

[67] Zhang X., Jin R., Zheng Y., Han D.-P., Chen K., Li J., Li H. (2020). Interactions between the enhanced recovery after surgery pathway and risk factors for lung infections after pulmonary malignancy operation. Transl. Lung Cancer Res..

